# Standardized fracture creation in the distal humerus and the olecranon for surgical training and biomechanical testing

**DOI:** 10.1007/s00402-021-04286-0

**Published:** 2022-01-01

**Authors:** Werner Schmoelz, Jan Philipp Zierleyn, Romed Hoermann, Rohit Arora

**Affiliations:** 1 Department of Orthopaedics and Traumatology, Medical University of Innsbruck, Anichstraße 35, 6020 Innsbruck, Austria; 2 Division Clinical and Functional Anatomy, Medical University of Innsbruck, Innsbruck, Austria

**Keywords:** Surgical training, Fracture simulation, Olecranon fracture, Distal humerus fracture

## Abstract

**Introduction:**

Surgical training and biomechanical testing require models that realistically represent the in vivo injury condition. The aim of this work was to develop and test a method for the generation of distal humerus fractures and olecranon fractures in human specimens, while preserving the soft tissue envelope.

**Methods:**

Twenty-one cadaveric upper extremity specimens (7 female, 14 male) were used. Two different experimental setups were developed, one to generate distal humerus fractures and one to generate olecranon fractures. Specimens were placed in a material testing machine and fractured with a predefined displacement. The force required for fracturing and the corresponding displacement were recorded and the induced energy was derived of the force–displacement graphs. After fracturing, CT imaging was performed and fractures were classified according to the AO classification.

**Results:**

Eleven distal humerus fractures and 10 olecranon fractures with intact soft tissue envelope could be created. Distal humerus fractures were classified as AO type C (*n* = 9) and as type B (*n* = 2), all olecranon fractures were classified as AO type B (*n* = 10). Distal humerus fractures required significantly more load than olecranon fractures (6077 N ± 1583 vs 4136 N ± 2368, *p* = 0.038) and absorbed more energy until fracture than olecranon fractures (17.8 J ± 9.1 vs 11.7 J ± 7.6, *p* = 0.11), while the displacement at fracture was similar (5.8 mm ± 1.6 vs 5.9 mm ± 3.1, *p* = 0.89).

**Conclusion:**

The experimental setups are suitable for generating olecranon fractures and distal humerus fractures with intact soft tissue mantle for surgical training and biomechanical testing.

## Introduction

In recent years, the training in orthopedic trauma surgery in the theatre was more complemented by hands on cadaver courses and virtual surgical simulation. Due to further specialization and complexity in surgical procedures and implants the professional education and teaching is evolving too. Thereby the traditional concept in surgical training of “see one, do one, teach one” [1] should be amended by hands on and simulation training to meet the demands of the public, modern medical systems and the economic pressure [1–3]. After residents first acquire basic surgical skills by taking an assisting role in a surgery, first hands on training and simulations can improve surgical and technical skills prior to performing an operation under supervision. In particular, because orthopedic trauma surgery require not only a competence in managing skeletal trauma but is often also heavily depending on technical skills [3]. There are several different ways to simulate a surgical procedure as realistically as possible such as computer-assisted, virtual reality models, bone surrogate models and cadaver workshops in which a surgery can be practiced on anatomical specimens [1, 4]. Different studies showed that skills acquired in simulated or hands on training can be transferred from the simulation to the surgery itself [5, 6]. With a focus on open reduction and internal fixation, Wegmann et al. [7] described a technique for fracture creation in the distal radius, by means of a drop test bench. Fresh frozen human forearms are positioned in a drop test bench to simulate a fall onto the outstretched arm. They reported real life clinical fracture patterns with an intact soft tissue mantle. This allows training of fracture treatment including practicing the surgical access with real life haptic and anatomical specimens. Wegmann et al. [7, 8] hypothesized that teaching with pre-fractured fresh frozen anatomical specimens is superior and enables a much closer training of real life situations in cadaver surgery workshops. A feedback survey of a cadaver workshop to improve the operation skills for distal radius fractures received an excellent feedback from the participants [8]. With a drop test bench, they also managed to fracture anatomical specimens for different body sides including the distal humerus and the proximal forearm [7–11]. An alternative setup to fracture creation with a drop test bench could be a setup in a material testing machine. A test setup for pre-fracturing anatomical specimens in a material testing machine will open up new possibilities, because in contrast to drop test benches, material testing machines are available in many institutions and laboratories. Standardized and reproducible fracture creation in anatomical specimen with intact soft tissue envelope could not only be used for surgical training but also for biomechanical investigations of different osteosyntheses techniques and implants. Up to now, the vast majority of comparative biomechanical studies of different osteosyntheses techniques and implants create fracture models by osteotomies [12, 13]. While they are well standardized and reproducible, they often only approximate real life fracture patters.

Therefore, the aim of the present study was to develop a test setup for creation of standardized and reproducible fractures of the olecranon and distal humerus with an intact soft tissue mantle for surgical teaching of residents and for training of surgeons to further improve their skills in more complex and challenging fractures [14]. Additionally, these fractures shall be evaluated for their potential use in biomechanical testing.

## Materials and methods

Twenty-one alcohol-glycerine-fixed cadaveric upper extremities provided by the Anatomy department of the Medical University of Innsbruck were used. The specimens were harvested from body donors, who had given their written consent for their bodies to be used for scientific and educational purposes. Inclusion criteria for the specimens were no bone injuries or surgical treatment regarding the cubital joint, no rheumatic diseases and an intact soft tissue mantle. The inclusion criteria were controlled for by fluoroscopy and visual inspection. The mean age of the donors was 74 (range 50–96). Seven upper extremities were from female body donors and fourteen from male body donors. Nine of the upper extremities were left/right paired and three were singular.

### Specimen preparation and experimental setup

**Fig. 1:**
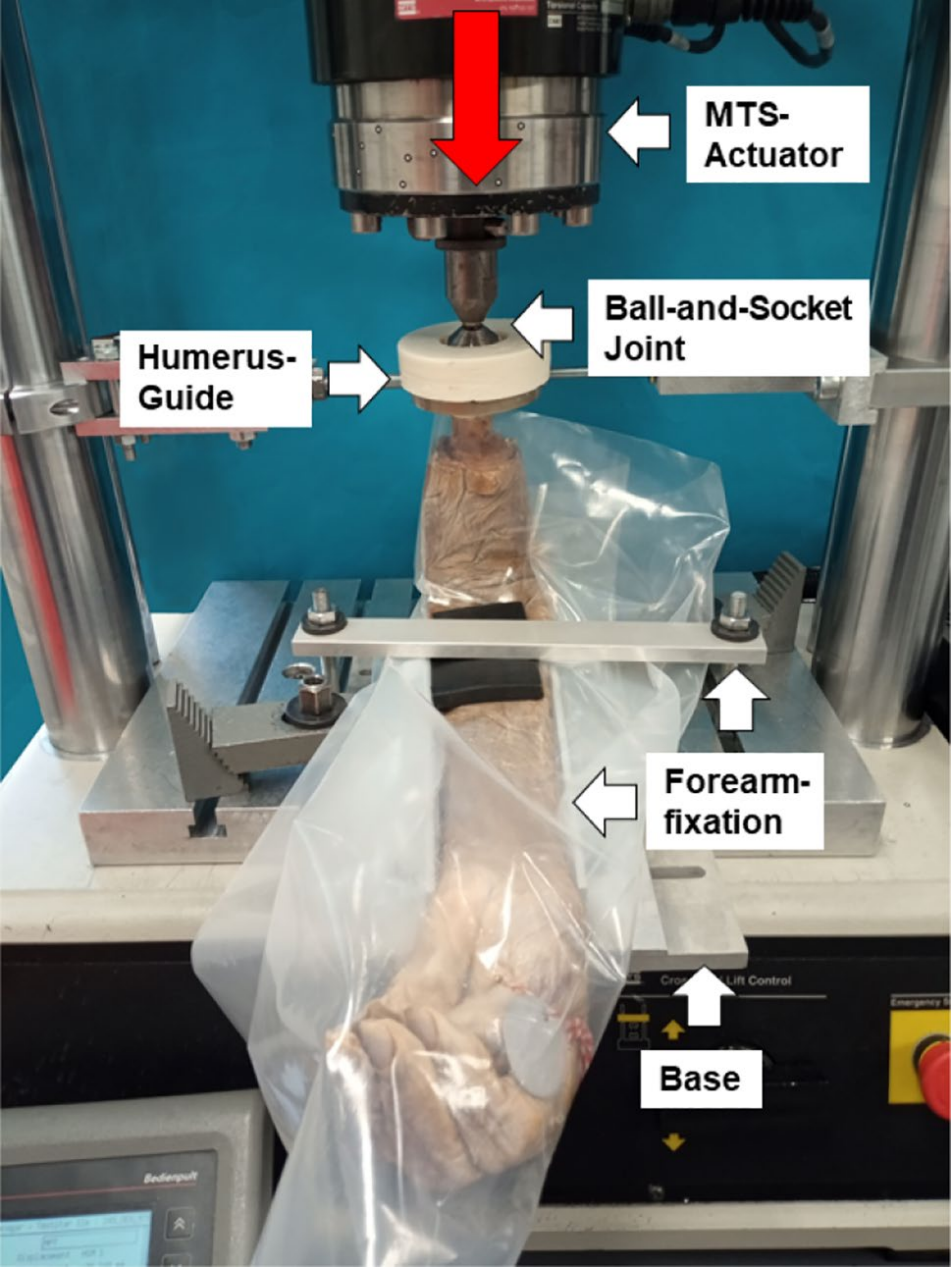
Setup of specimen loading in a servo-hydraulic material testing machine. The red arrow indicates the load application

In a first step, the humeri were prepared to a standardized length of 20 cm from the distal end and the proximal 5 cm of the prepared humeri were freed from soft tissue. In the second step, the proximal humeri (approx. 3.5 cm) were embedded in epoxy resin (RenCast FC 53 NB + filler DT082 Huntsman, The Woodlands, TX, USA) by use of a purpose-build cylindrical mold. During the embedding process, the longitudinal axis of the humeri was aligned to the axis of the cylindrical embedding Two different experimental setups to create either a humerus fracture or an olecranon fracture were realized in a biaxial servo‐hydraulic material testing machine (MTS Mini-Bionix II 858; MTS, Eden Prairie, MN, USA) (Fig. 1). In both setups, the proximal part of the humerus, embedded in epoxy resin, was placed in a guide that allowed movement along the vertical axis and rotation around it, while the forearm was rigidly fixed to the load frame. The displacement induced by the testing machine to fracture either distal humerus or olecranon was transmitted from the actuator of the testing machine to the specimen via a ball and socket joint between the actuator and the proximal part of the embedded humerus.

**Fig. 2:**
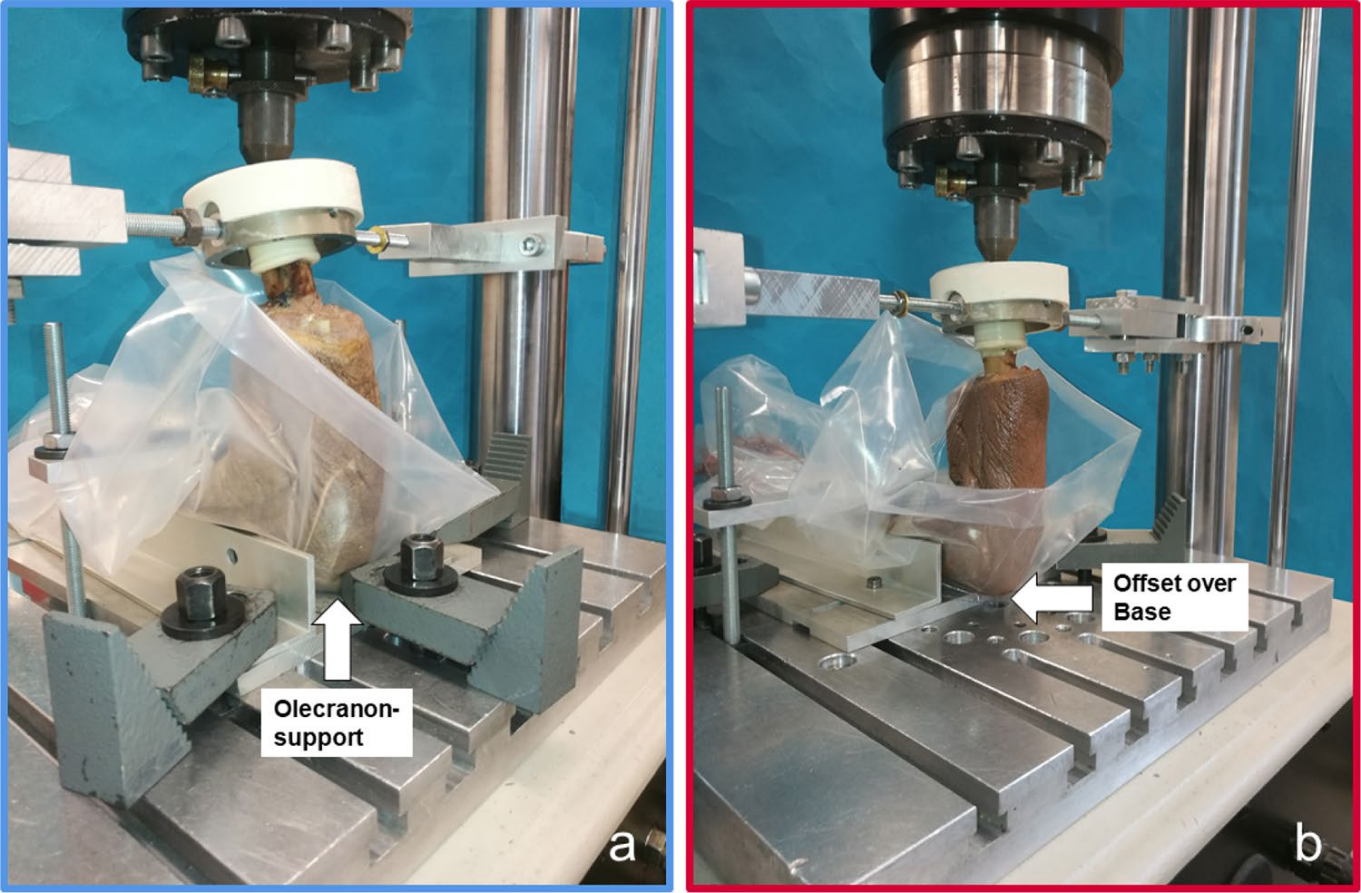
Close up view of the setup for specimen fixation in a load frame for generation of humerus fractures (**a**) and of olecranon fractures (**b**)

To create a humerus fracture the cubital joint was positioned to approximately 105° flexion and a mechanical stop was placed at the olecranon to prevent slippage and fracture of the olecranon (Fig. 2a). To provoke a fracture of the olecranon, the angle in the cubital joint was set to 90° flexion and the olecranon was positioned in the load frame of the material testing machine with an offset of approx. 15 mm over the base plate to create a predetermined breaking point (Fig. 2b).

For fracture creation, the specimens were subjected to a preload of 50 N followed by an impact of 10 mm (displacement controlled ramp with 100 mm/s). After loading, the fracture was verified by fluoroscopy. If no fracture was detected in the fluoroscopy, image loading was repeated with a higher impact displacement (15 mm). During the experiment, the displacement of the actuator, associated force and time were recorded with a sample rate of 1000 Hz.

Subsequent to fracture verification by fluoroscopy, a clinical CT (LightSpeed VCT 16, GE Healthcare, Chicago, USA) was conducted and one observer classified the fractures according to the current AO-classification system and Mayo classification [15, 16]. To measure the trabecular bone mineral density (BMD) of the distal radius based on a combination of the techniques described by Burt et al. [17] and Krappinger et al. [18] was used. In the region of interest described by Burt et al. [17], three measurements were performed similar to Krappinger et al. [18].

The maximum force occurring during fracture and the corresponding displacement were derived of the force–displacement graphs. The energy absorbed by the test objects until fracture was calculated by numerical integration of the force–displacement curve. After maximum force, the plots showed a steep drop, therefore, the energy absorption was calculated up to the displacement occurring at the maximum applied force.

### Statistical analysis

A *t* test was used for comparison of the maximum force, energy and displacement of olecranon and humerus fractures. In the SPSS software package (IBM SPSS Statistics, Version 24.0.0.1, IBM Corporation, Armonk, New York, USA), a Pearson correlation was used to examine the correlation of the measured BMD and the maximum applied force, energy and displacement.

## Results

With the setup designed for distal humerus fractures in 11 of the 15 loaded specimens the desired fracture could be created (73%), in 4 specimens loading in the distal humerus setup resulted in an olecranon fracture. With the setup intended for olecranon fractures, the desired fracture type could be created in all specimens (100%).

**Fig. 3:**
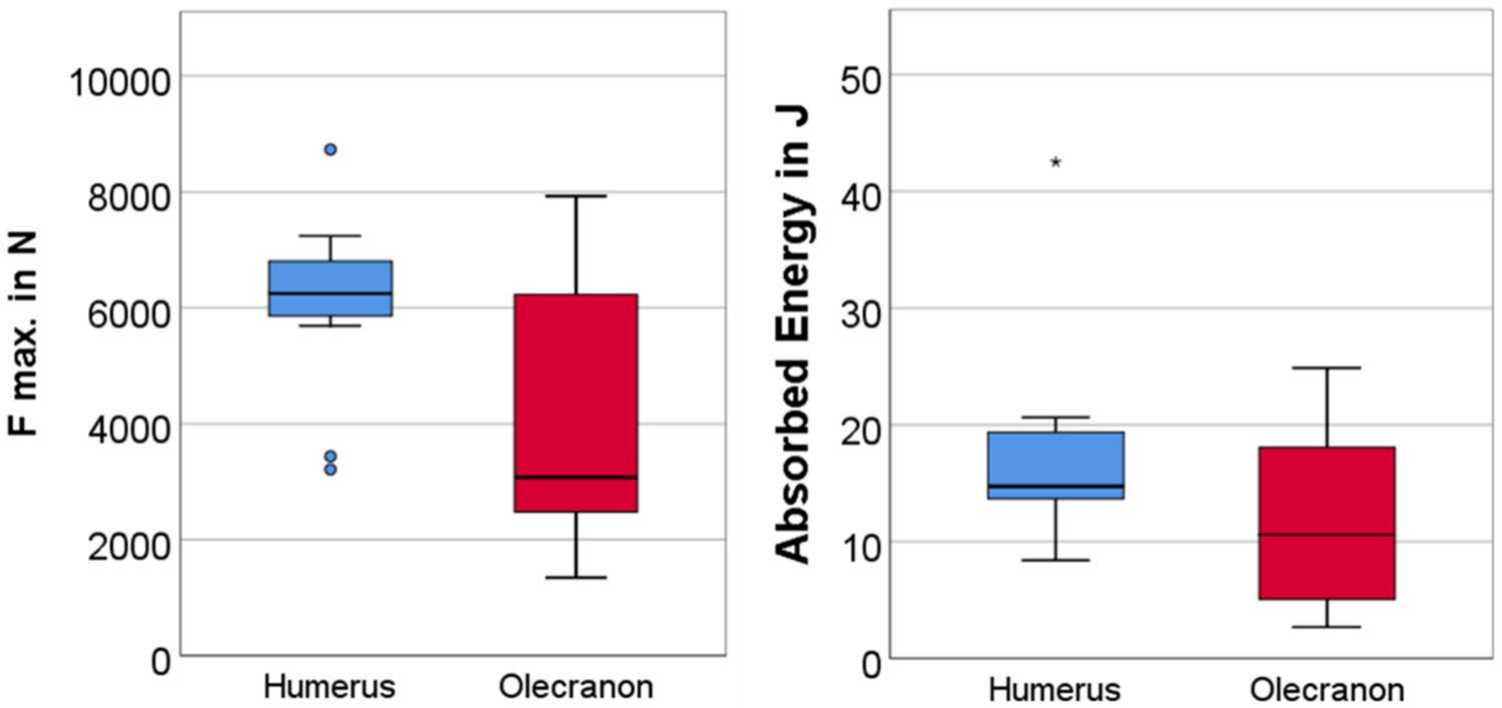
Boxplot of maximum force at fracture and absorbed energy until fracture

Fractures of the distal humerus showed a significant higher maximum force than fractures of the olecranon (6077 N ± 1583 vs 4136 N ± 2368; *p* = 0.038). The corresponding displacement at maximum force for the humerus fractures was 5.8 mm ± 1.6 mm and 5.9 mm ± 3.1 mm for the olecranon fracture (*p* = 0.89). The energy absorption until maximum fracture load was 17.8 J ± 9.1 for distal humerus fractures versus 11.7 J ± 7.6 fore olecranon fractures (*p* = 0.11) (Tables 1, 2; Fig. 3).

**Table 1:** Overview of specimens with humerus fractures

Humerus fractures					
Specimen	AO fracture classification	Max. force (N)	Displacement at max force (mm)	Energy (J)	Tb. BMD (mg/cm^3)^
AG101L	13B1.3	6477	6.0	20.6	97
AG105L	13B2.3	5692	5.9	14.7	118
AG107R	13C1.1	6177	4.7	14.1	141
AG106R	13C2.2	6249	4.5	14.7	103
AG101R	13C3.1	6614	6.5	19.6	117
AG107L	13C3.1	6025	3.9	13.3	138
AG109R	13C3.1	3438	6.5	9.9	27
AG102L	13C3.2	7243	7.4	19.1	147
AG104R	13C3.2	8732	9.5	42.5	177
AG106L	13C3.2	6993	5.9	18.2	121
AG109L	13C3.2	3211	4.6	8.4	34
Mean ± SD		6077 ± 1583	5.8 ± 1.6	17.8 ± 9.1	111 ± 46

**Table 2:** Overview of specimens with olecranon fractures

Olecranon fractures						
Specimen	AO fracture classification	Mayo classification type	Max. force (N)	Displacement at max. force (mm)	Energy (J)	Tb. BMD (mg/cm^3)^
AG103R	2U1B1	II B	2504	5.0	7.9	145
AG104L	2U1B1	II B	7927	5.8	24.9	49
AG105R	2U1B1	II B	6131	8.1	18.6	78
AG108R	2U1B1	II B	2583	3.5	5.1	146
AG111R	2U1B1	I A	6231	6.8	16.6	207
AG102R	2U1B1	II B	6720	3.9	13.3	165
AG103L	2U1B1	II B	1348	3.4	2.7	69
AG108L	2U1B1	I B	1868	3.6	3.3	178
AG110L	2U1B1	II B	3566	13.6	18.1	112
AG112R	2U1B1	II B	2478	5.8	6.9	102
Mean ± SD			4136 ± 2368	5.9 ± 3.1	11.7 ± 7.6	125 ± 52

**Fig. 4:**
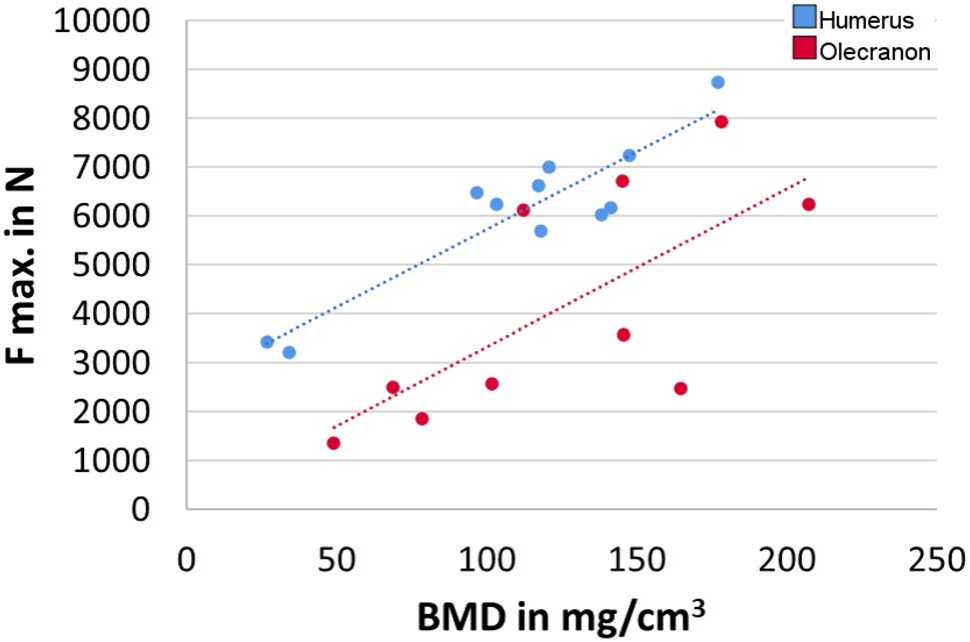
Correlation of BMD with maximum force at fracture

The average trabecular BMD of the distal radius of the specimens was 118 mg/cm^3^ ± 48. Correlating the BMD with parameters of the fracture creation showed a significant correlation with the maximum load (*R* = 0.92, *p* < 0.0001) and absorbed energy (*R* = 0.677, *p* = 0.022) for distal humerus fractures and with the maximal load (*R* = 0.701, *p* = 0.024) and absorbed energy (*R* = 0.691, *p* = 0.027) for olecranon fractures (Fig. 4). No correlation with displacement was found for either humerus fractures (*R* = 0.337, *p* = 0.31) or olecranon fractures (*R* = 0.387, *p* = 0.269).

**Fig. 5:**
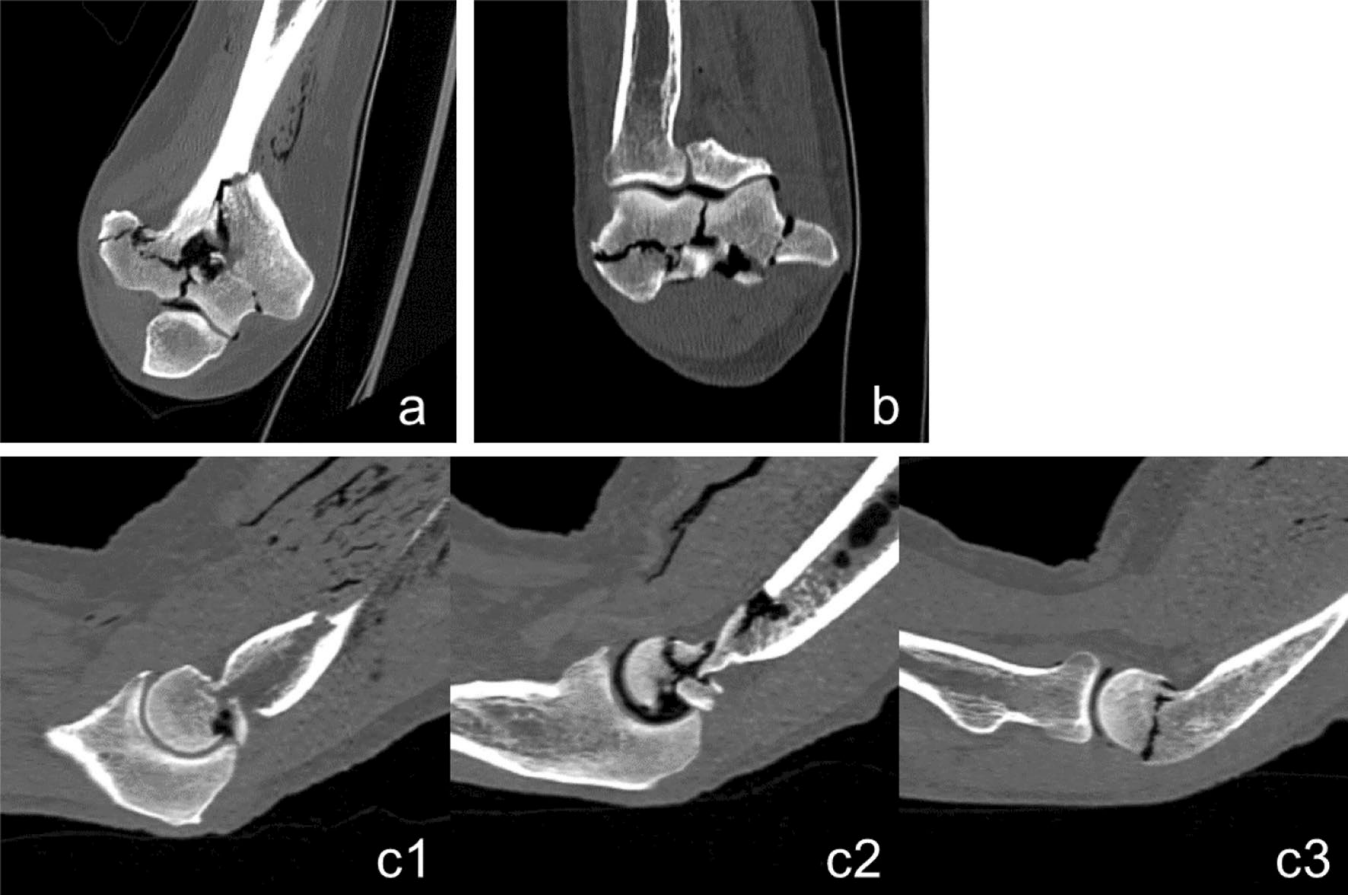
Exemplary CT cross sections of a typical humerus fracture pattern (AO-classification 13C3.2) **a** coronal plane, **b** axial plane, **c1–c3** sagittal plane

Regarding the fracture classification according to the AO, 8 of the 11 distal humerus fractures showed an AO type C fracture with a similar fracture pattern (Fig. 5). Thereby, the medial part of the condyle was separated by a fracture parallel to the longitudinal axis through middle of epiphysis and metaphysis, extending up to the transition to the diaphysis. Additionally, a second transvers fracture line, running just above or below the transcondylar axis separated the capitulum humeri from the metaphysis in most cases. In some of these cases, the transverse fracture line also separated the trochlea humeri from the before described medial fragment. Two of the distal humerus fractures were classified as AO type B fractures, one with the capitulum humeri and one with the trochlear humeri staying intact. One more showed a complete articular fracture with a fracture pattern that presents in shape of a capital T in coronal plane with the cross at the transition from metaphysis to diaphysis.

**Fig. 6:**
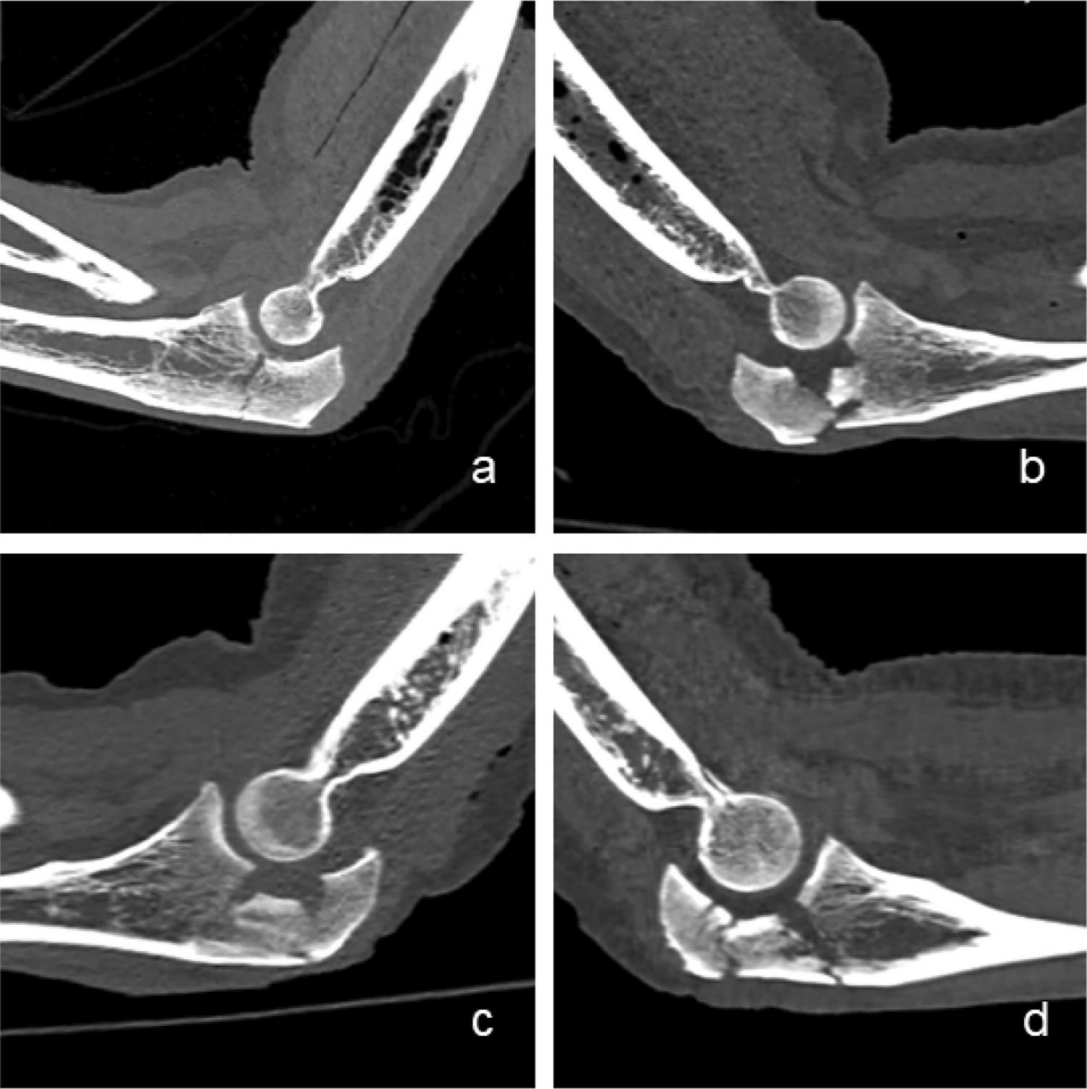
Exemplary CT cross sections illustrating the heterogenic fracture patterns of four different olecranon fractures (AO-classification **a–d** U1B1, Mayo classification **a** type I A, **b–d** type II B)

All olecranon fractures were classified as AO type B fractures. In all fractures, the olecranon was separated from the ulna by a fracture line running through the bare area or slightly proximal. Additionally, the olecrani were fragmented in varying manners and showed a heterogenic fracture pattern (Fig. 6). One olecranon fracture was a simple olecranon fracture, while nine were multi-fragmental fractures. Four showed a distinct impression of the articular surface. According to the Mayo classification, eight olecranon fractures were classified as type II B, one as type I A and one as type I B.

## Discussion

The study shows that with the chosen experimental setups, it was possible to selectively produce either fractures of distal humerus or olecranon with an intact soft tissue mantle. The skin showed only some minor signs of injury manifested by pressure marks and small cuts similar as they can occur in real life injuries. These minor skin injuries still allow the training of the surgical access for the fracture treatment.

Of the two fracture types, the setup for olecranon fractures worked particularly well and generated the desired fracture type in all the specimens it was applied to (100%). The radiological assessment of olecranon fractures showed variable fracture patterns. This might be due to anatomical variations in size and shape of the used specimens. A more uniform fracture pattern with less complex two part fracture pattern might be achieved by increasing the cubital flexion angle and apply the loading with a stepwise increased displacement until a fracture is present.

With the experimental setup for generation of distal humerus fractures, the desired fracture could be generated in 11 of 15 specimens (73%). In 4 of the 15 trials, olecranon fractures instead of intended humerus fractures were created (27%). The unintended olecranon fractures with the setup for the distal humerus tended to be more complex and likely pose a greater challenge for surgical treatment in training courses. The success rate for distal humerus fractures might be improved, by changing the support and mechanical stop of olecranon to prevent the dislocation during fracture generation to a custom fit support and fixation of the olecranon (e.g. a plaster cast, or mold with epoxy resin).

Considering the different success rate in fracture creation and cross-overs of the distal humerus and olecranon fractures, if an equal number of the two fracture locations is desired, it is recommended to start with distal humerus fractures.

The aim of the study was to develop and design experimental setups capable of reproducibly generating the desired fracture type with realistic fracture patterns and mechanism. Comparing the fracture mechanism and fracture pattern of the distal humerus fractures with clinical studies showed a good agreement for distal humerus fractures [19, 20].

The created olecranon fractures show comparable fracture patterns as reported in the literature for complex olecranon fractures [21]. However, in an epidemiological study on ulna fractures, Duckworth et al. [22] observed a higher percentage of less complex and simpler two part fractures of the olecranon than created with the presented setup. A less complex two-part fracture pattern might be achieved by increasing the cubital flexion angle and reducing the displacement applied for fracture creation.

The trabecular BMD of the distal radius in the fractured specimens correlated well with the maximum fracture load and the absorbed energy until fracture. This is in accordance to the reports in the literature reporting the bone morphology of the distal radius being a good predictor for fragility fractures [23]. Recently, Marcoin et al. fractured isolated distal humeri for a biomechanical study and also reported a correlation between the fracture load and BMD [24].

Using pre-fractured specimens for surgical training courses has a more than 20-year-long tradition at our institution. Initially, forearm fractures were created with manual hydraulic devices, followed by manual fracture creation in a material testing machine up to the current setup with a simulated impact with a servo-hydraulic material machine. In the literature, fracture creation in anatomical specimens is described either by dropping a dead weight or by the use of a material testing machine [7, 9–11, 25–27]. In drop dead weight, fracture generation specimens are subjected to an impact with a predefined amount of energy with an optional limit of the displacement, while in material testing machines, specimens are subjected to an impact with a predefined displacements or force with an unlimited amount of energy. Therefore, a comparison of the applied energy for fracture creation in drop dead weight tests and material testing machines are not very meaningful. E.g Wegmann et al. [7] reported an average impact of 134.7 J for fracture creation in distal radius specimens, while in the current study, the energy to fracture either the distal humerus or the olecranon was substantially less. This can be explained by the fact, that for material testing machine setups usually the energy absorbed by the specimen until fracture is calculated with the applied force and displacement, while in drop dead weights setups, the impact energy is calculated with the gravity force of the dead weights and their drop height. However, parts of the input energy in drop dead weight tests might also be absorbed by the mechanical stop limiting the displacement or the soft tissue. To allow an energy comparison of the two test setups, the energy, the applied force and displacement until fracture in drop dead weight tests should be recorded and used to calculate the absorbed energy of the specimen until fracture.

Another possible application for pre-fractured specimens could be in biomechanical testing and comparison of different fracture stabilizations. While the presence of real life fractures is a crucial factor in surgical training, it is also important in the field of biomechanical testing and comparison of different fracture stabilizations. Up to now, the standard for fracture simulation in biomechanical testing is the creation of a fracture by standardized osteotomies [12, 13]. This allows the comparison of different fracture stabilization treatments in comparable instabilities. However, with a standardized and reproducible technique to create real life fracture patterns in specimens, biomechanical studies on fracture stabilizations will also move from idealized fracture patterns closer to the real life applications of the fracture stabilization techniques. The designed and tested setups for fracture generation in the present study resulted in a variation of real life fracture patterns. Therefore, prior to their application for biomechanical comparison of fracture stabilization techniques, the setups will have to be further optimized to narrow down the variation in fracture patterns to limit the number specimens required to obtain comparable test groups for comparison of fracture treatment techniques.

A limitation of the present study is the use of alcohol-glycerine fixed cadaveric upper extremities for the experiments. However, it can be assumed that the setups for fracture creation of distal humerus and olecranon fractures will work equally well with fresh frozen specimens, only the maximum force until fracture and its corresponding displacement as well as the required energy until fracture might vary slightly [28].

Concluding the designed and tested setups for fracture creation with an intact soft tissue envelope in distal humerus fractures and olecranon fractures are capable of producing a range of real life fracture types and patterns for surgical training courses, while for biomechanical testing and comparison of fracture stabilization procedures, a more homogenized fracture pattern would be desirable.
